# The Cross-Cultured Validity and Reliability of the Vietnamese Nijmegen Questionnaire

**DOI:** 10.7759/cureus.78709

**Published:** 2025-02-07

**Authors:** Ngoc-Minh Nguyen, Hanh Thi Bich Tran, Thi-Quynh-Nhu Do, Philippe Fait, Gregory Reychler

**Affiliations:** 1 Department of Rehabilitation, Nursing and Medical Technology Faculty, University of Medicine and Pharmacy at Ho Chi Minh City, Ho Chi Minh, VNM; 2 Institute of Experimental and Clinical Research (IREC), Pôle de Pneumologie, ORL et Dermatologie, Université Catholique de Louvain, Bruxelles, BEL; 3 College of Nursing and Health Sciences, Flinders University, Adelaide, AUS; 4 Rehabilitation Consultant, Vietnam Assistance for the Handicapped (VNAH), Hà Nội, VNM; 5 Department of Human Kinetics, University of Quebec at Trois-Rivières, Trois-Rivières, CAN; 6 Research Group on Neuromusculoskeletal Disorders (GRAN), University of Quebec at Trois-Rivières, Trois-Rivières, CAN; 7 Department of Physiotherapy, Physio Interactive and Cortex Clinic, Quebec City, CAN

**Keywords:** cross-cultured validity, hyperventilation syndrome, nijmegen questionnaire, structural validity, test–re-test reliability

## Abstract

Introduction: The Nijmegen Questionnaire assesses hyperventilation syndrome (HVS), but a validated Vietnamese version is lacking. This study investigates the cross-culture validity, structural validity, and reliability of the Vietnamese Nijmegen questionnaire (VNQ) for screening HVS in primary healthcare settings.

Methods: Following Beaton's guidelines, translation and adaptation involved two independent expert panels for content validity. Exploratory factor analysis (EFA) and confirmatory factor analysis (CFA) examined the VNQ's structure. Reliability was assessed via Cronbach's alpha, intraclass correlation coefficients, Bland-Altman analysis, and linear regression.

Results: Content validity was excellent (all I-CVIs > 0.79 and all S-CVIs > 0.8). EFA revealed a four-factor structure, including the original three NQ factors and a new “psychology” factor, confirmed by CFA. Cronbach's alpha and ICC values exceeded 0.7 and 0.8, respectively, indicating good internal consistency and test-retest reliability. Bland-Altman analysis showed low systematic error, and linear regression revealed no proportional bias (P > 0.05).

Conclusion: The VNQ demonstrates excellent content validity, acceptable structural validity, and reliable psychometric properties for HVS screening in primary healthcare settings.

## Introduction

Dysfunctional breathing significantly deteriorates health conditions [1], with hyperventilation syndrome (HVS) being the most prevalent type. This latter manifests through a wide spectrum of symptoms such as stress, depression, anxiety, chest pain, and light-headedness, which collectively impair the quality of life (QoL) [2]. These symptoms are often variable, nonspecific, and overlap with many other diseases, complicating the diagnosis process [3]. The physiological basis of these symptoms is believed to be a decrease in arterial carbon dioxide due to excessive alveolar ventilation exacerbated by psychological aspects such as anxiety, and stress- and anxiety-related problems [4]. Despite its heavy impact on QoL and an estimated prevalence of around 20% [5], HVS remains an underrecognized condition. Enhanced screening is thus essential for timely appropriate treatment and early rehabilitation [3], especially given the increased prevalence of HVS post-COVID-19 pandemic [6].

Traditionally, HVS diagnosis involves the exclusion of other organic diseases, posing additional challenges to HVS screening [7]. Moreover, HVS can occur in people without underlying disease [5]. Delayed diagnosis can exacerbate symptoms of HVS leading to conditions such as tetany [8]. Currently, there is no gold standard for HVS diagnosis, but a combination of tools is recommended, including the Nijmegen questionnaire (NQ), end-tidal CO2 (PETCO2) measurement, holding breath test (HBT), hyperventilation provocation test (HVPT), and cardio-pulmonary exercise test (CPET) [9].

The NQ stands out as a practical tool due to its simplicity and proven validity [10]. Compared to other tools, the NQ has several advantages. The NQ is a simple questionnaire with only 16 items, reducing the time required for assessment without incurring costs. Additionally, it can be self-administered by individuals with HVS. Moreover, the NQ has high sensitivity (86%-91%) and specificity (79%-92%), depending on health conditions and cut-off threshold [11]. Additionally, the NQ is suitable for screening HVS in healthy populations [5]. Despite its proven utility, the NQ has not yet been translated into Vietnamese. This study aims to translate the NQ into Vietnamese and to examine its cross-cultural validity, structural validity, and reliability, enabling its use for screening HVS in primary healthcare settings throughout Vietnam.

## Materials and methods

Ethics statement

The study was approved by the Ethics Committee at the University of Medicine and Pharmacy at Ho Chi Minh City (approval No. 1187 HĐĐĐ-ĐHYD). All participants were fully informed about the study protocol and provided written informed consent. Participant information will be stored securely and accessible only with a password. All stages of the study were conducted following the Declaration of Helsinki and Clinical Good Practice guidelines.

Participants

Participants were randomly recruited in the community and were required to be literate in Vietnamese. All participants were over 18 years old and had the appropriate consciousness level to answer the questionnaire.

The Nijmegen questionnaire

The NQ rates 16 HVS-related symptoms on a 5-point scale across three factors: shortness of breath (items 1, 2, 6, 7, 8, 11, 15), peripheral tetany (items 10, 12, 13, 14), and central tetany (items 1, 3, 4, 5, 9), with a total possible score of 64 [12].

Translation and crossed-culture validity

The translation and cross-cultural validity of the NQ were conducted following the COnsensus-based Standards for the selection of health status Measurement Instruments (COSMIN) guidelines [13,14]. First, permission was obtained from the original authors of the NQ, which is free to use without copyright restrictions [10]. Then, the translation and back-translation processes adhered to the guidelines for the Process of Cross-Cultural Adaptation of Self-Report Measures [15]. Two translators initially translated the NQ into Vietnamese. A review meeting involving these translators and an expert was held to synthesize the Vietnamese version of the NQ. The VNQ was then back-translated by two different translators. Experts reviewed both the original and back-translated versions to create a synthesized back-translated NQ (B-NQ). The VNQ and B-NQ were reviewed by a committee of five experts, whose feedback was used to finalize the VNQ. A different panel of five experts subsequently assessed the content validity of the VNQ. The clarity and relevance of the VNQ were rated on a 4-point Likert scale (from 1: cannot be used, not relevant/clear to 4: highly relevant/clear). Feedback from the expert panel was collected and incorporated to refine the translated version before final approval [16].

The face validity of the VNQ was conducted with 30 participants [14]. Participants rated the comprehensibility of the NQ from 1 (unable to understand) to 4 (easy to understand). Their feedback was used to make further modifications to VNQ.

Structural validity

To examine the structural validity of the VNQ, exploratory factor analysis (EFA) was conducted to either confirm the original three-factor structure (shortness of breath, peripheral tetany, and central tetany) or establish a new structure [10]. This was followed by a confirmatory factor analysis (CFA) to assess the structural validity of the VNQ using the original or newly identified factor model [14].

Reliability

The internal consistency and test-retest reliability of the final VNQ were evaluated with a sample of at least 50 participants [14]. The VNQ was distributed via email or Google Forms, and participants completed the questionnaire twice within a two-week interval. Participants also completed the 11-point Global Rating of Change (GRC) during the second administration to detect any changes in health status. The GRC is a visual analog scale for recalling the health status at different time points. Participants with a non-zero GRC score were excluded from the reliability analysis [17].

Data analysis

Cross-Culture Validity

Content validity was assessed using the item content validity index (I-CVI) and scale-level content validity index (S-CVI) [18]. The I-CVI was calculated by dividing the number of experts rating an item as relevant/clear (score 3 or 4) by the total number of experts [16]. An I-ICV score greater than 0.79 is considered excellent, between 0.7 and 0.79 is average, and less than 0.7 is poor [16]. The S-CVI was evaluated through two indices: Universal Agreement among experts (S-CVI/UA) and Average CVI (S-CVI/Ave) [16]. The S-CVI/UA is the ratio of items with an I-CVI of 1 to the total number of items [16]. Content validity is considered excellent if S-CVI/UA ≥ 0.8, and S-CVI/Ave ≥ 0.9 [16]. The risk chance agreement was assessed using the Kappa statistic, with values > 0.74 being excellent, 0.6-0.74 good, and 0.4-0.59 fair [16]. The face validity of the VNQ was examined by having 30 participants rate the comprehensibility of the questionnaire.

Structural Validity

The EFA was conducted using the Kaiser-Meyer-Olkin (KMO) measure of sampling adequacy and Bartlett's test of sphericity to confirm the assumptions [19]. The KMO value assessed the sampling adequacy, while Bartlett's test evaluated the presence of sufficient correlations among the items to proceed with the factor analysis. The rotation of sums of squared loadings was used to determine the total variance explained by each factor. Factor loadings above 0.4 were considered acceptable.

The CFA was performed to evaluate the model fit of the VNQ, using indicators such as the Chi-squared, Comparative Fit Index (CFI), Root Mean Square Error of Approximation (RMSEA), and Standardized Root Mean Square Residual (SRMSR). These model fit indices were assessed based on the COSMIN guidelines and general recommendations for CFA [14]. For the CFI, values of 0.95 or higher are considered to indicate good model fit, while values between 0.90 and 0.95 suggest adequate fit, and below 0.90 indicate poor fit [20]. For the RMSEA, values of 0.05 or lower suggest a good model fit, and values up to 0.08 are considered acceptable [20]. Values above 0.10 indicate poor fit. For the SRMSR, values of 0.08 or lower are considered a good model fit [20].

Internal Consistency

The internal consistency of each exploratory factor was evaluated using Cronbach's Alpha [21]. An alpha value above 0.7 indicates acceptable internal consistency [21].

Test-Retest Reliability

Test-retest reliability was analyzed using a two-way mixed-effects absolute agreement intraclass correlation coefficient (ICC) [22]. The ICCs were calculated for the total score as well as for the confirmed factors. An ICC above 0.5 is considered acceptable, above 0.8 indicates good reliability, and above 0.9 is excellent [23]. To assess bias and limits of agreement, a Bland-Altman plot was utilized [24]. Proportional bias was further investigated using linear regression [25].

The statistical analyses were performed using SPSS software, while the CFA was assessed using AMOS software (Released 2023. IBM SPSS Statistics for Windows, Version 29.0.2.0. IBM Corp., Armonk, NY).

## Results

Participants

A total of 82 participants (40 females and 42 males) were recruited for the study. None reported any cardiopulmonary or health issues within the preceding six weeks. Thirty participants were involved solely in the face validity investigation. Fifty-five participants were recruited to investigate test-retest reliability, but three participants were excluded due to changes in the GRC score, leaving 52 participants for the reliability assessment. Participants’ ages ranged from 24 to 59 years.

Translation

A free translation method was suggested for the “feeling tense” item, to describe a stuffy feeling in Vietnamese. The expert panel recommended removing “deeper” from the “Faster/Deeper breathing” item to avoid possible confusion with the “unable to breathe deeply” item. The VNQ was ultimately accepted (supplemental table).

Content validity

The content validity of the VNQ was found to be excellent overall (Table 1). The clarity I-CVI for the “Feeling tense” item was poor (0.4), while all other I-CVIs for clarity and relevance were above 0.79. All items' modified Kappa statistic coefficient was above 0.74, except for the clarity of the “Feeling tense” item (0.37). All S-CVIs were all above 0.8.

**Table 1:** The content validity of the Vietnamese NQ A: The number of experts rated the item as a 3 or 4 on a 4-point scale; I-CVI: Item-content validity index; S-CVI: Scale-level-content validity index; S-CVI/UA: universal agreement among experts; S-CVI/Ave is averages of the item-level CVIs; Pc: Probability of chance agreement; K: Modified kappa statistic coefficient.

Item	Clarity				Relevance			
	A	I-CVI	Pc	K	A	I-CVI	Pc	K
Chest pain	5/5	1.00	0.03	1.00	5/5	1.00	0.03	1.00
Feeling tense	2/5	0.4	0.05	0.37	4/5	0.80	0.16	0.76
Blurred vision	5/5	1.00	0.03	1.00	5/5	1.00	0.03	1.00
Dizzy spells	5/5	1.00	0.03	1.00	5/5	1.00	0.03	1.00
Feeling confused	5/5	1.00	0.03	1.00	5/5	1.00	0.03	1.00
Faster breathing	5/5	1.00	0.03	1.00	4/5	1.00	0.03	1.00
Short of breath	5/5	1.00	0.03	1.00	5/5	1.00	0.03	1.00
Tight feelings in the chest	5/5	1.00	0.03	1.00	5/5	1.00	0.03	1.00
Bloated feeling in the stomach	4/5	0.80	0.16	0.76	4/5	0.80	0.16	0.76
Tingling fingers	5/5	1.00	0.03	1.00	5/5	1.00	0.03	1.00
Unable to breathe deeply	5/5	1.00	0.03	1.00	5/5	1.00	0.03	1.00
Stiff fingers or arms	5/5	1.00	0.03	1.00	5/5	1.00	0.03	1.00
Tight feelings around the mouth	5/5	1.00	0.03	1.00	5/5	1.00	0.03	1.00
Cold hands or feet	5/5	1.00	0.03	1.00	5/5	1.00	0.03	1.00
Palpitations	5/5	1.00	0.03	1.00	5/5	1.00	0.03	1.00
Feelings of anxiety	5/5	1.00	0.03	1.00	4/5	1.00	0.16	0.76
	S-CVI/UA = 0.88; S-CVI/Ave = 0.95				S-CVI/UA = 0.88; S-CVI/Ave = 0.98			

Face validity

Thirty participants rated the ease of understanding of the VNQ. None rated it at levels 1 and 2. Seven participants (23%) rated it at level 3, and 23 participants (77%) rated it at level 4.

Structural validity

The KMO measure of sampling adequacy had a value of 0.795, and Bartlett's test of sphericity was statistically significant (p < 0.001), indicating that the assumptions were met to proceed with the EFA. The eigenvalues suggested the presence of four factors. Accordingly, the researchers proposed adding a “psychology” factor as a fourth latent factor for the VNQ, besides the three original factors. For each latent factor, any items with a factor loading below 0.4 were removed. The total variance explained by each factor and the rotated component matrix are presented in Table 2.

**Table 2:** Rotated factor matrix with Kaiser normalization and variance explained for each factor Extraction Method: Principal Factor Analysis. Rotation Method: Varimax with Kaiser Normalization. ^a^Rotation converged in 6 iterations.

	Rotated factor matrix ^a^					
Items			Factor			
			Short of breath	Peripheral tetany	Central tetany	Psychology
11	Unable to breathe deeply		0.815	NA	NA	NA
6	Faster breathing		0.783	NA	NA	NA
7	Short of breath		0.522	NA	NA	NA
2	Felling tense		0.500	NA	NA	0.482
15	Palpitations		0.482	NA	NA	0.504
5	Feeling confused		0.420	NA	0.476	NA
12	Stiff fingers or arms		NA	0.738	NA	NA
13	Tight feelings around the mouth		NA	0.706	NA	NA
10	Tingling fingers		NA	0.624	NA	NA
8	Tight feelings in the chest		NA	0.612	NA	NA
14	Cold hands or feet		NA	0.608	NA	0.404
1	Chest pain		NA	NA	0.818	NA
3	Blurred vision		NA	NA	0.787	NA
4	Dizzy spells		NA	NA	0.560	NA
9	Bloated feeling in the stomach		NA	NA	NA	0.833
16	Feelings of anxiety		NA	NA	NA	0.784
Rotation Sums of Squared Loadings		% variance explained	17.618	16.602	14.736	14.078
		Cumulative %	63.034			

**Figure 1:**
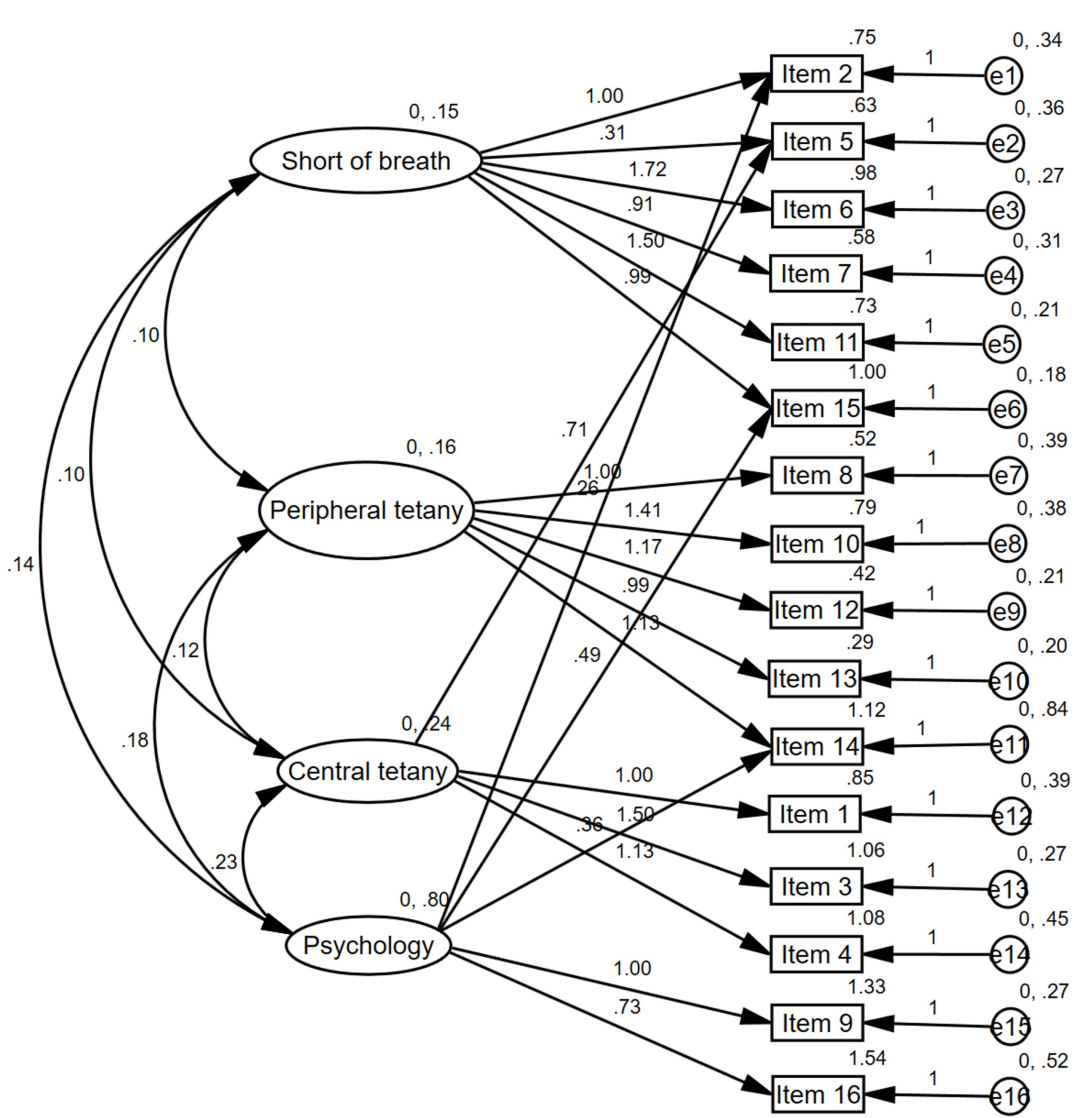
Structural validity for the Vietnamese Nijmegen questionnaire with four factors solution

The structural validity of the VNQ was evaluated using CFA (Figure 1). Based on the four-factor structure established with EFA, the results showed that the factor loadings ranged from 0.31 to 1.72 for short of breath factor, 0.90 to 1.49 for peripheral tetany factor, 0.24 to 1.50 for central tetany, and 0.26 to 1.00 for psychology factor. The model fit indices suggested that the model fit the data well, with a Chi-squared of 104.6 (p = 0.2), CFI of 0.96, RMSEA of 0.04, and SRMSR of 0.0785.

Reliability

The internal consistency and test-retest reliability of the VNQ were evaluated using data from 52 participants. The Cronbach’s Alpha value for the total score and the individual factors were all above 0.7 (Table 3), indicating good internal consistency. The correlation between each factor and the total score exceeded 0.8. 

**Table 3:** Internal consistency and test-retest reliability for the VNQ VNQ: Vietnamese Nijmegen questionnaire, ICC: Intraclass Correlation Coefficient, IC: Interval Confidence *: p-value < 0.001

	Correlation	Cronbach’s α	ICC	95% IC
Total		0.89	0.93*	0.87 – 0.96
Shortness of breath	0.89	0.82	0.91*	0.84 – 0.95
Peripheral tetany	0.82	0.74	0.87*	0.77 – 0.92
Central tetany	0.78	0.75	0.85*	0.74 – 0.92
Psychology	0.89	0.80	0.93*	0.89 – 0.96

**Figure 2:**
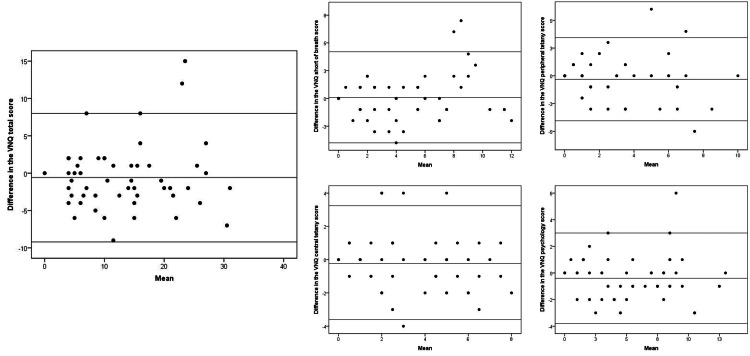
Bland-Altman plot of total and factor scores for the Vietnamese Nijmegen questionnaire Solid lines represent bias mean. Upper and lower dashed lines represent the upper and lower limits of agreement.

The test-retest reliability analysis revealed statistically significant ICCs above 0.8 for the total score and factor scores, suggesting good reliability (Table 3). The Bland-Altman analysis showed a bias ranging from -0.58 to 0.08 for the total and factor scores (Table 4, Figure 2). The standard error measurement (SEM) was reported as 0.61 for the total VNQ, 0.29 for shortness of breath, 0.26 for peripheral tetany, 0.24 for central tetany, and 0.24 for psychology. Linear regression analysis demonstrated no proportional bias, with all p-values above 0.05 (Table 4).

**Table 4:** Bias and limit of agreement for test-retest reliability for Vietnamese Nijmegen questionnaire

Limit of agreement	Bias	Upper	Lower	P-value of linear regression
Total	-0.58	8.1	-9.2	0.54
Shortness of breath	0.08	4.20	-4.04	0.22
Peripheral tetany	-0.31	3.43	-4.05	0.10
Central tetany	-0.23	3.14	-3.60	0.10
Psychology	-0.40	3.00	-3.80	0.65

## Discussion

Due to the unavailability of gold measurements for HVS [4,9], and the lack of guidance for HSV diagnosis in Vietnam, this study investigated the crossed-culture (content, face) and structural validity. The translation process of the VNQ strictly adhered to Beaton's guidelines. This study employed two independent expert panels: one to collect opinions and modify the VNQ, and another to investigate content validity. Based on the first panel's recommendations, minor changes were made to the questionnaire. The second expert panel then evaluated the content validity. This approach enhanced the objective assessment of content validity.

The content validity results indicated an excellent corresponding index. Only the “feeling tense” item had a poor I-CVI for clarity, while the other I-CVIs were excellent in both clarity and relevance [16]. The overall content validity of the questionnaire was excellent, with both S-CVI/UA and S-CVI/Ave for clarity and relevance above 0.8 and 0.9, respectively [16]. Compared to a critical review of the NQ, our study reported better content validity than the original study [26]. This may be due to a better understanding of HVS and the NQ. This study also reported good face validity, with all the participants rating the VNQ as understandable or easy to understand. According to our search, this is the first study to report face validity through the easy-to-understand level from a target population. This result was not surprising since the questionnaire only included symptom listing without any complex medical terminologies or ambiguous sentences.

The EFA results showed that the hypothesized three-factor structure of the original NQ was not replicated in the VNQ. The result suggested the presence of a fourth latent factor. This fourth factor included five explained items (feeling tense, bloated feeling in the stomach, cold hands or feet, palpitations, and feelings of anxiety), which the researchers name the “psychology” factor. The new factor finding is not surprising considering that HVS is commonly accompanied by psychological symptoms such as anxiety, and panic [27]. This finding is supported by another study that also detected new factors compared to the original version [28], suggesting the cross-cultural adaptations of questionnaires may reveal new factors that better fit the target population. Additionally, the cumulative explained variance was 64%, higher than that reported in two validation studies of the NQ for patients with asthma (54% to 58%), potentially attributable to the influence of the disease characteristics [28,29]. The results of the CFA showed that the four-factor model, consisting of the three original factors of the NQ (shortness of breath, peripheral tetany, and central tetany) and an additional “psychology” factor, provided a good fit model to the observed data for the VNQ. The factor loadings for the four factors were acceptable [20]. The model fit indices, including a Chi-squared, CFI, RMSEA, and SRMSR, all indicated a good fit for the model [20], supporting the four-factor structure of the VNQ.

The reliability analysis showed excellent results, with very high Cronbach’s Alpha values for total score and factor scores. All factor values exceeded the acceptable level of 0.7, and the total score was good (> 0.8) [21]. This study reported similar internal consistency results to the original study [12], though the high number of items raises the potential risk of high-value bias [14].

This study also reported excellent ICC values for absolute agreement for the total score and good ICC values for each factor [23]. The results were comparable to another study [30]. To enhance the accuracy of reliability assessment as required by the COSMIN [13,14], the study used the GRC despite the potential risk of “recall bias” associated with this method [17]. The Bland-Altman analysis demonstrated a low systematic error, with biases of -0.58 for the total score, 0.08 for shortness of breath, -0.31 for peripheral tetany, -0.23 for central tetany, and -0.4 for psychology. The random errors were relatively wide for the total score (-9 to 8), and narrower for each factor, suggesting a cautious interpretation of VNQ total score changes in the healthy population. The Bland-Altman plots indicated that the increased mean difference does not change the test-retest difference [24]. Additionally, the statistically non-significant result of the linear regression analysis rejected a proportional bias [14].

This study employed a rigorous methodology, featuring two expert panels for assessing content validity. Furthermore, the structural validity analysis identified a new latent psychology factor, potentially improving targeted interventions for individuals with HVS. The study also applied control tests when assessing reliability, enhancing confidence in the results. However, the small sample size limited conclusions about parameter estimates and construct validity. Additionally, the study was conducted on participants without reported health issues, so caution is warranted when applying the VNQ to other populations, such as those with asthma or other chronic lung diseases.

## Conclusions

The VNQ has excellent content validity, as demonstrated by the rigorous translation process and the thorough assessment by two expert panels who evaluated the clarity and relevance of the questionnaire items, thus enhancing the objectivity of the content validity assessment. The study also found the VNQ has acceptable structural validity, with a four-factor model fitting the data well and including an additional "psychology” factor not in the original NQ. This suggests the VNQ may better capture HVS's complexity. Additionally, the VNQ has reliable psychometric properties, with high internal consistency and test-retest reliability as evidenced by a controlled test, indicating a consistent measurement. These findings make the VNQ a valuable tool for primary healthcare providers in Vietnam to effectively screen and identify individuals with HVS, a crucial step in delivering targeted interventions and improving patient outcomes.

## Appendices

**Table 5:** Vietnamese Nijmegen Questionnaire

STT		Không bao giờ – 0	Hiếm khi - 1	Đôi khi - 2	Thường xuyên - 3	Rất thường xuyên - 4	Tổng
1	Đau ngực						
2	Cảm giác ngột ngạt/bí bách						
3	Nhìn mờ						
4	Có những cơn chóng mặt						
5	Cảm giác mơ hồ/lú lẫn						
6	Thở nhanh hơn						
7	Khó thở						
8	Cảm giác căng nặng ngực						
9	Cảm giác chướng bụng đầy hơi						
10	Các ngón tay bị châm chích/tê bì						
11	Không thể thở sâu						
12	Cứng ngón tay hoặc cả cánh tay						
13	Cảm giác căng ở xung quanh miệng						
14	Lạnh bàn tay hoặc bàn chân						
15	Đánh trống ngực / tim đập nhanh						
16	Cảm giác lo lắng						
Tổng điểm							
